# New Insights into Alterations in PL Proteins Affecting Their Binding to DNA after Exposure of *Mytilus galloprovincialis* to Mercury—A Possible Risk to Sperm Chromatin Structure?

**DOI:** 10.3390/ijms22115893

**Published:** 2021-05-31

**Authors:** Gennaro Lettieri, Rosaria Notariale, Nadia Carusone, Antonella Giarra, Marco Trifuoggi, Caterina Manna, Marina Piscopo

**Affiliations:** 1 Department of Biology, University of Naples Federico II, Via Cinthia, 21, 80126 Naples, Italy; gennarole@outlook.com (G.L.); nadia.carusone@libero.it (N.C.); 2 Department of Precision Medicine, School of Medicine, University of Campania “Luigi Vanvitelli”, via Luigi de Crecchio, 80138 Naples, Italy; notarialer@gmail.com (R.N.); caterina.manna@unicampania.it (C.M.); 3 Department of Chemical Sciences, University of Naples Federico II, Via Cinthia, 21, 80126 Naples, Italy; antonella.giarra@unina.it (A.G.); marco.trifuoggi@unina.it (M.T.)

**Keywords:** mussel, HgCl_2_, spermatozoa, PL proteins, sperm nuclei, fluorescence spectroscopy, turbidity assays, reproduction

## Abstract

Mercury (Hg) is a highly toxic and widespread pollutant. We previously reported that the exposure of *Mytilus galloprovincialis* for 24 h to doses of HgCl_2_ similar to those found in seawater (range 1–100 pM) produced alterations in the properties of protamine-like (PL) proteins that rendered them unable to bind and protect DNA from oxidative damage. In the present work, to deepen our studies, we analyzed PL proteins by turbidimetry and fluorescence spectroscopy and performed salt-induced release analyses of these proteins from sperm nuclei after the exposure of mussels to HgCl2 at the same doses. Turbidity assays indicated that mercury, at these doses, induced PL protein aggregates, whereas fluorescence spectroscopy measurements showed mercury-induced conformational changes. Indeed, the mobility of the PLII band changed in sodium dodecyl sulphate-polyacrylamide gel electrophoresis, particularly after exposure to 10-pM HgCl_2_, confirming the mercury-induced structural rearrangement. Finally, exposure to HgCl_2_ at all doses produced alterations in PL-DNA binding, detectable by DNA absorption spectra after the PL protein addition and by a decreased release of PLII and PLIII from the sperm nuclei. In conclusion, in this paper, we reported Hg-induced PL protein alterations that could adversely affect mussel reproductive activity, providing an insight into the molecular mechanism of Hg-related infertility.

## 1. Introduction

### 1.1. Environmental Pollution by Mercury

Technological progress and industrialization have produced the release of several pollutants in aquatic and terrestrial environments. Among all pollutants, heavy metals are receiving a lot of attention from environmental scientists because of their toxic natures. Although heavy metals are generally present in trace amounts in natural waters, a large number of them are toxic even at very low concentrations [1], posing a serious danger to marine organisms [2,3,4]. Among the various heavy metals, mercury (Hg) has drawn a lot of attention, as it is the most toxic nonessential metal [5]. Mercury has captured the attention of the scientific community since the 1950s, when the first evidence of hazardous environmental impacts were reported [6], which led to policies and regulations to limit Hg emissions. The World Health Organization (WHO), in 2013, classified mercury as one of the 10 chemicals of greatest public health concern worldwide.

### 1.2. Seawater Mercury Pollution

Seawater mercury contamination is a problem for the environment and human health [7]. Indeed, in the aquatic cycle, this metal be transformed into MeHg by anaerobic bacteria [8]. Indeed, more importantly, the concentrations of Hg species in seawater are very low, about 1 pM [9], but these levels are enough for driving the bioaccumulation and biomagnification of this toxic metal, along the aquatic food chain up to humans [10,11]. Furthermore, mercury is well-studied in terms of its bioavailability, bioaccumulation, biomagnification and cellular toxicity, overall, in bivalves [12].

### 1.3. Mytilus galloprovincialis as Sentinel Organism

The value of bivalve mollusks and, particularly, mussels (*Mytilus* spp.) for use as sentinel organisms for pollution monitoring in the coastal environments has been well-established in several experimental and field studies [13]. The mussel *M. galloprovincialis*, for its wide geographic distribution, abundance, easy accessibility and other important characteristics, is commonly employed as model in the field of environmental toxicology [14,15] and considered an ideal bioindicator of pollutants on the Mediterranean coasts. This filter-feeding intertidal invertebrate, in fact, responds quickly to environmental pollution and accumulates high levels of various types of contaminants [16,17], providing a time-integrated measure of environmental contamination [18,19]. 

### 1.4. Mercury and Mytilus galloprovincialis Reproduction

In the bivalve life cycle, the early developmental stages prove to be the most responsive to several contaminants, including heavy metals [20], pesticides [21] and antifouling paints [22], and the concentrations of heavy metals capable of causing lethal toxicity in embryos and larvae have been shown to be considerably lower than the ones that are lethal to adults [23,24]. In particular, mercury was also found to be more toxic than some other metals, like lead (Pb) and cadmium (Cd), to *M. galloprovincialis* embryos [25], and Beiras et al. 1995 [26] indicated a consistent decreased sensitivity to this metal as the *M. galloprovincialis* developmental stages increased in age. We have previously shown alterations in the properties of protamine-like proteins (PL) of *M. galloprovincialis* after exposure to sublethal doses of metals such as copper and cadmium [3,4,27]. Recently, we showed that the exposure of *M. galloprovincialis* for 24 h to 1, 10 and 100 pM HgCl_2_ caused protamine-like (PL) proteins that were partly in the form of aggregates and were unable to bind and protect DNA from oxidative damage [28,29]. These exposure doses reflected the amount of mercury present in the waters of the Mediterranean basin and the North Atlantic oceans [9,30,31]. We found these results to be extremely interesting and worthy of further investigation given the paucity of information in the literature on reproductive health effects in *M. galloprovincialis* following exposure to picomolar doses of mercury chloride. Therefore, in the present work, we expanded our investigations in order to understand the nature of mercury-induced alterations in PL proteins, affecting them binding with DNA. Specifically, we analyzed, by turbidity assays, the effect of adding increasing concentrations of HgCl_2_, in the range of 1–100 pM, to PL proteins and evaluated, by fluorescence spectroscopy, alterations in PL proteins from mussels exposed to 1, 10 and 100-pM HgCl_2_. Finally, we performed salt-induced release analyses of these proteins from sperm nuclei after exposure of mussels to 1, 10 and 100-pM HgCl_2_. 

## 2. Results

### 2.1. Turbidity Assays on PL Proteins

**Figure 1:**
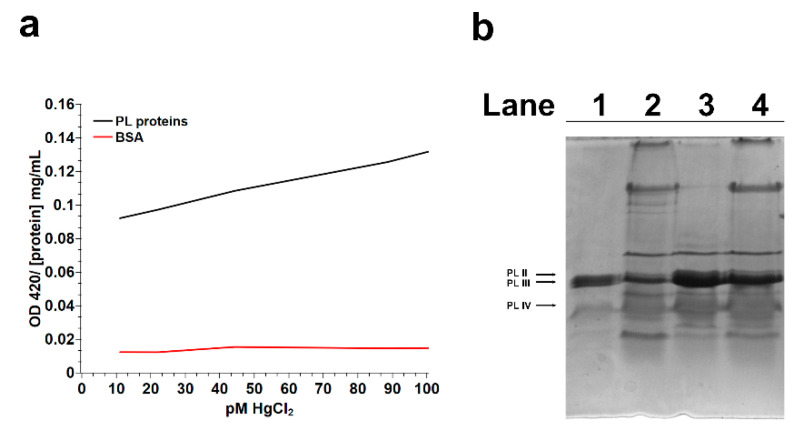
Turbidity assay (**a**) following 420-nm absorbance changes as a function of HgCl_2_ adding: in black, the trend of PL proteins, and, in red, that of BSA (negative control). An electrophoretic analysis by SDS PAGE (**b**) of *M. galloprovincialis* PL proteins extracted from nonexposed (lane 1) and exposed mussels (lanes 2, 3 and 4): to 1, 10 and 100-pM HgCl_2_, respectively. (**b**) A reused figure that corresponds to (B) of Figure 3 (Lettieri et al. 2021) obtained under the Creative Common CC BY license. Lettieri G, Notariale R, Ambrosino A, Di Bonito A, Giarra A, Trifuoggi M, Manna C, Piscopo M. Spermatozoa Transcriptional Response and Alterations in PL Proteins Properties after Exposure of *M. galloprovincialis* to Mercury. *Int J Mol Sci.* 2021 Feb 5;22(4):1618. doi: 10.3390/ijms22041618.

Figure 1a shows the results of the turbidity assay obtained by adding increasing amounts of HgCl_2_, in the range of 1–100 pM, to a solution of PL proteins extracted from nonexposed mussels and measuring the changes of 420-nm absorbance. The graph describing the results obtained indicated that the absorbance at 420 nm of the PL solution slightly increased as the concentration of HgCl_2_ incremented differently from that of Bovine Serum Albumin (BSA) (Figure 1a). This result suggests the possible formation of protein aggregates, corroborating that already obtained in Sodium Dodecyl Sulphate-Polyacrylamide Gel Electrophoresis (SDS-PAGE) (Figure 1b) [28], in which additional protein bands with reduced mobility were observed in the samples of PL proteins extracted from mussels exposed to 1, 10 and 100-pM HgCl_2_. 

### 2.2. Fluorescence Spectroscopy

**Figure 2:**
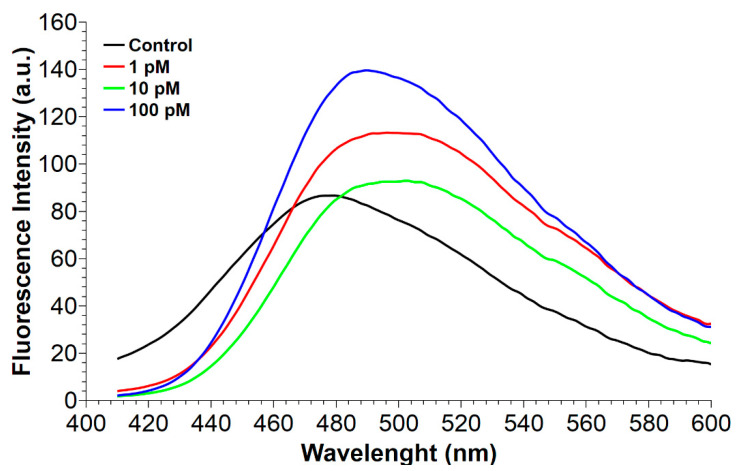
Fluorescence spectra of PL proteins from nonexposed (control) and HgCl_2_-exposed mussels.

In order to investigate the mercury-induced structural changes of PL proteins, fluorescence spectra at an excitation wavelength of 350 nm were performed on these proteins from nonexposed mussels (control) and exposed to HgCl_2_ using a hydrophobic dye such as 8-Anilinonaphthalene-1-sulfonic acid (ANS) [32]. The spectra of PL from nonexposed mussels presented a peak at 479 nm, while those of mussels exposed to all HgCl_2_ doses showed a red shift respectively of 17.5 nm (peak at 496.5 nm, 1 pM), 23.5 nm (peak at 502.5 nm, 10 pM) and 11.0 nm (peak at 490.0 nm, 100 pM) (Figure 2). The red shift shown could be compatible with the binding of ANS to more polar regions of these proteins in accordance with those reported by Bothra et al. 1998 [33]. In addition, an increase in the ANS fluorescence intensity of about 1.3 and 1.6 times for PL from mussels exposed to 1 and 100-pM HgCl_2_ respectively, was observed (Figure 2). These differences indicated alterations in the conformation of PL proteins from HgCl_2_-exposed mussels.

### 2.3. SDS-PAGE Analysis of PL Proteins

**Figure 3:**
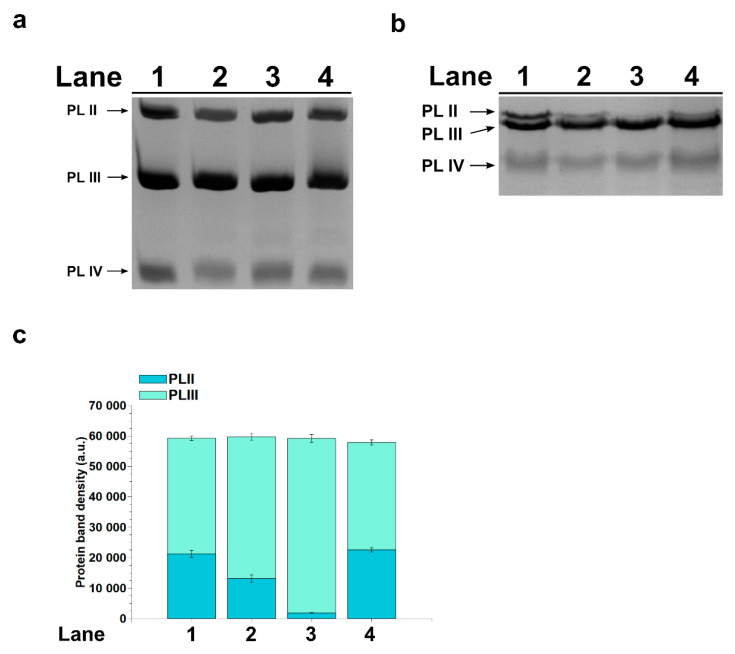
SDS-PAGE (**b**) and densitometric bands analyses (**c**) of the PLII and PLIII proteins, extracted from spermatozoa of nonexposed mussels (lane 1) and of mussels exposed to 1, 10 and 100-pM HgCl_2_ (lanes 2, 3 and 4, respectively). AU-PAGE (**a**) of the PL proteins from the spermatozoa of nonexposed mussels (lane 1) and of mussels exposed to 1, 10 and 100-pM HgCl_2_ (lanes 2, 3 and 4, respectively) (*n* = 6).

In order to identify any changes in the PL protein electrophoretic mobility after the exposure of mussels to HgCl_2_, we performed an electrophoretic analysis by SDS-PAGE, maintaining the same final percentage of acrylamide described by Lettieri et al. 2021 [28] but changing the Acrylamide-Bisacrylamide ratio (29:1) in order to obtain an higher resolution of the protein bands. In these conditions, the electrophoretic pattern by SDS-PAGE of the PLII and PLIII proteins (Figure 3) showed that after the exposure of mussels to 10-pM HgCl_2_ (Figure 3b, lane 3), the band corresponding to the PLII protein did not seem to appear on the gel in the position observed in the control (Figure 3b, lane 1). This behavior was, in part, also observed in the PL samples from mussels exposed to 1 and 100-pM HgCl_2_, in which a lower amount of PLII was revealed in comparison with nonexposed mussels (Figure 3b, lanes 2 and 4). Differently, the electrophoretic analysis carried out on the same sample by AU-PAGE showed a complete PL pattern, including PLII proteins (Figure 3a). These results suggested that mercury could influence the electrophoretic mobility of the PLII protein in the presence of SDS. The densitometric analysis performed on the electrophoretic bands (Figure 3c) in SDS-PAGE showed that the amount of protein corresponding to the apparent PLIII band in lane 3 was higher with respect to that measured for the PLIII protein in the samples loaded in the other lanes and similar to the sum of the bands relative to the PLII and PLIII proteins. This result supports the hypothesis of a comigration of the PLII and PLIII proteins in SDS-PAGE in the sample obtained from mussels exposed to 10-pM HgCl_2_. 

### 2.4. DNA Absorption Spectrum after PL Proteins Addition

**Figure 4:**
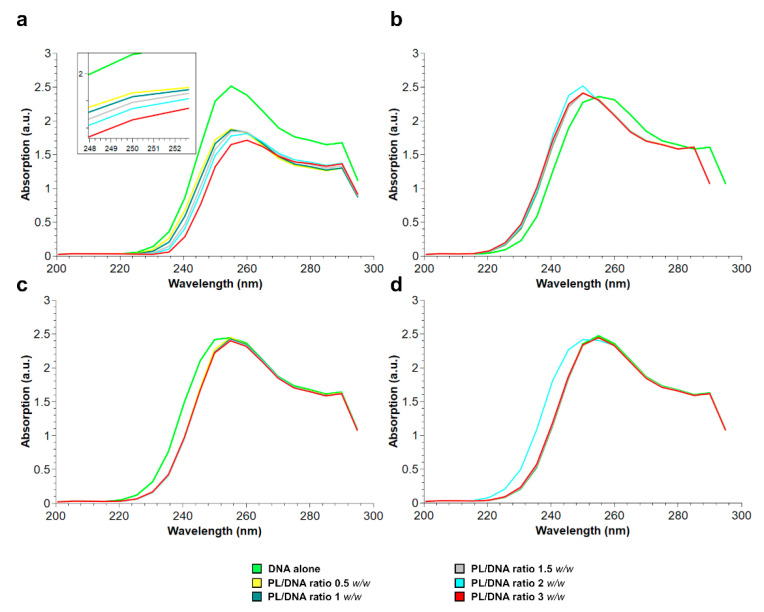
Absorption spectra in the range of 200–300 nm of plasmid DNA alone and PL/DNA mixtures at various PL/DNA ratios: PL extracted from nonexposed mussels (**a**) and PL extracted from mussels exposed to 1 pM (**b**), 10 pM (**c**) and 100-pM HgCl_2_ (**d**), respectively. Green: plasmid alone, yellow: PL/DNA 0.5 ratio (*w/w*), blue: PL/DNA ratio 1 (*w/w*), grey: PL/DNA ratio 1.5 (*w/w*), blue: PL/DNA ratio 2 (*w/w*) and red: PL/DNA ratio 3 (*w/w*).

In order to evaluate possible effects of the exposure of mussels to HgCl_2_ on the ability of PL proteins to bind to DNA, the absorption spectrum in the 200–300-nm range of plasmid DNA alone was compared with the absorption spectra of the same following the addition of increasing amounts of PL (0.5–1–1.5–2–3 PL/DNA (*w/w*) ratios) from nonexposed and exposed HgCl_2_ mussels. Figure 4 shows the results of these assays. The decrease of the maximum DNA absorption shown in Figure 4a is indicative that PL extracted from nonexposed mussels binds DNA. Contrarily, adding PL extracted from mussels exposed to all the HgCl_2_ doses did not cause changes in the DNA absorption spectrum. In fact, in these cases, the absorption spectra of plasmid DNA alone and plasmid DNA in the presence of increasing amounts of PL are almost superimposable. All this could suggest any alterations in the PL–DNA binding.

### 2.5. Release of PL Proteins from Sperm Nuclei

**Figure 5:**
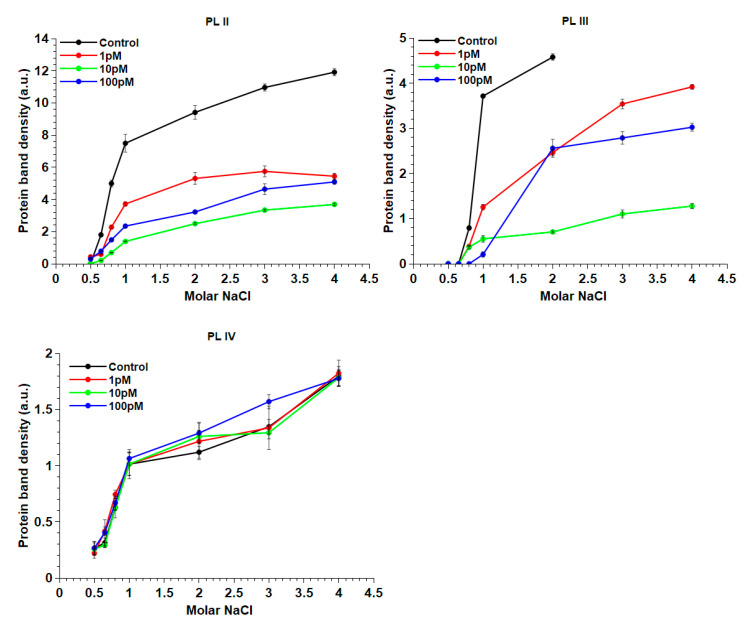
The release of PL from sperm nuclei at different NaCl molar concentrations in the control mussels and exposed to 1, 10 and 100-pM HgCl_2_ (*n* = 6).

In order to confirm possible changes in the PL–DNA binding of mussels exposed to HgCl_2_ at the three doses, an analysis of the release of these proteins from the sperm nuclei with increasing concentrations of NaCl was performed as shown in Figure 5. No significant differences in the PLIV release were observed in the control and HgCl_2_-exposed mussels, while alterations were showed both for PLII and PLIII. In particular, PLII was released in smaller amounts after exposure of the mussels to all three doses of HgCl_2_. About 50% of this protein was released after the exposure of mussels to 1 and 100-pM HgCl_2_, while only about 33% after exposure to 10-pM HgCl_2_. PLIII release from the sperm nuclei of mussel nonexposed ended at 2-M NaCl. The further addition of NaCl did not release this protein. In fact, the electrophoretic analysis of the proteins release at 3 and 4-M NaCl did not show the band corresponding to this protein. For this reason, in the graph, there are not values of a densitometric analysis for these latter concentrations of NaCl. Regarding PLIII, after the exposure of mussels to 1 pM, a larger amount of NaCl (4 M instead of 2 M) was required for obtaining about 80% of this protein in comparison with the control condition. The exposure of mussels to 100-pM HgCl_2_ instead caused a PLIII release of only about 55% at 2-M NaCl, and the latter additions of NaCl did not significantly increase the amount released of this protein. Finally, only 28% of PLIII was released after the exposure of mussels to 10-pM HgCl_2_. 

## 3. Discussion

Considering the extensive exposure of organisms and the well-recognized toxicity of mercury that causes, even with low-level exposure, several negative impacts on different biological functions, in this work, we deepened our studies on the effects of this metal on the reproductive health of *M. galloprovincialis*, having already reported not only alterations in the expression of some gonadal stress genes but, also, on the properties of the PL proteins, which represent the main nuclear basic protein component of the sperm chromatin of this organism [28]. First, we confirmed our hypothesis that mercury could induce PL protein aggregate formation. Indeed, turbidity assays showed that the addition of increasing concentrations of HgCl_2_, in the range of 1–100 pM, to PL proteins from nonexposed mussels caused a slight increase in the absorbance at 420 nm, indicative of protein aggregate formation. That HgCl_2_ induces protein aggregation has already been reported for other proteins. For example, Arnhold et al. 2015 [34] identified amyloid protein aggregation in the cell nucleus as a novel Hg–bio-interaction pathway [34], and we, in our previous studies, showed the formation of a mercerized tetrameric Hb as a result of treating human erythrocytes with increasing concentrations of HgCl_2_ [5]. We also investigated the possibility of mercury-induced conformational changes in PL proteins using fluorescence spectroscopy. The fluorescence analyses indicated conformational changes in PL proteins extracted from mussel exposed to HgCl_2_, because for these proteins, an increase in ANS fluorescence was observed. This effect was particularly relevant for PL proteins after the exposure of mussels to 1 and 100-pM HgCl_2_ and suggested a likely rearrangement of the proteins that produced an increase in the hydrophobic surface area available for ANS binding. It is well-known that both proteins and DNA undergo conformational changes to form functional complexes and, also, to facilitate interactions with other molecules. These modifications have a direct consequence on the stability and specificity of the complex, as well as the cooperativity of interactions between multiple entities. Generally, the proteins with the most conformational changes are those that have the most contact with DNA. In general, these proteins are those that have a lower dipole moment but a more positive net charge at the interface, just like PL proteins. Some hydrophobic residues such as Cys, Ala and Gly undergo much more conformational adaptations at the interface than others [35], and the two latter amino acids are particularly abundant in PL proteins. As is well-known, mercury is capable of binding cysteines [28] and histidines [36]. The binding of mercury ions to these amino acids may underlie the conformational changes we observed from the fluorescence analyses, but we cannot exclude that mercury could also be able to bind other amino acids in which PL proteins are richer, such as glycine and alanine [37]. Furthermore, the increase in fluorescence and, especially, the red shift of the emission maximum wavelength of ANS presented by the PL proteins obtained from mussels exposed to HgCl_2_ confirmed the interactions between these proteins and the fluorescent probe. We can hypothesize that the exposure of mussels to HgCl_2_ could cause a lower degree of compactness of these proteins, exposing more of their hydrophobic groups. This structural rearrangement might be possible for this type of proteins, because it has been shown that small proteins with a high number of hydrophilic residues can have a higher hydrodynamic radius than larger ones [38]. Thus, perturbations at the polar surface can result in a more compact protein, and this explains the faster migration observed in the SDS-PAGE of the PLII protein isolated from mussels exposed to HgCl_2_. Interestingly, this behavior, under our experimental conditions, was particularly noticeable after exposure of the mussels to 10-pM HgCl_2_ compared to when they were exposed to 1 and 100-pM HgCl_2_. This result could suggest a possible hormesis effect. Hormesis is a dose/response characterized by a biphasic effect. Many organisms/biological systems exposed to a broad range of stressors exhibit different responses depending on the dose, and various examples of hormesis effects have been reported in the literature [39,40,41,42]. The faster migration of the PLII protein, observed in the SDS-PAGE, was already reported for this protein after the exposure of *M. galloprovincialis* to CdCl_2_ [43]. All these effects could be the consequence of the well-known observation that chromatin-associated proteins are possible targets of mercury ion toxicity, because studies of rodent tissue culture cells exposed to HgCl_2_ have shown that mercury ions concentrate in cell nuclei and associate with chromatin [44]. In addition, it is well-known that Hg ions can also modulate the activity of certain proteins by changing their conformation. This has been demonstrated, for example, for the bacterial homodimer metalloregulator MerR, which represses transcription in the absence of mercury and activates transcription upon binding to Hg(II) [45], and for the dual-function transcription factor MerR from *B. megaterium*, whose apo- and Hg2+-bound conformations act as repressor and activator, respectively [46]. The conformational changes in PL proteins therefore prompted us to investigate whether the exposure of mussels to HgCl_2_ could affect their ability to bind DNA. After all, we had already shown, by EMSA, a decreased DNA-binding capacity of the PL proteins from HgCl_2_-exposed mussels for the pGEM3 DNA plasmid [28]. In the present work, we demonstrated that the absorption spectra of plasmid DNA alone and plasmid DNA in the presence of increasing amounts of PL proteins from mussels exposed to 1, 10 and 100-pM HgCl_2_ were almost superimposed. This behavior could indicate either that the proteins were unable to bind to DNA or that their binding to DNA was different, and to investigate this in more detail, we carried out analyses of the release of PL proteins from sperm nuclei. This analysis showed that PLIV was released in the same manner in nonexposed and HgCl_2_-exposed mussels. Contrarily, relevant changes were observed both for PLII and PLIII. In particular, these two proteins were released in lower amounts from the sperm nuclei of mussels exposed to all HgCl_2_ doses, a situation that we had already found after the exposure of *M. galloprovincialis* to copper chloride. [27]. Interestingly, even with this type of analysis, the 10-picomolar HgCl_2_ exposure dose was the one that gave the greatest differences. In fact, this dose of HgCl_2_ was the one that caused the lowest release of PLII and PLIII, which turned out to be around 33% and 28% for PLII and PLIII, suggesting a stronger DNA binding of PL proteins under this HgCl_2_ exposure condition. Therefore, our results revealed a different DNA binding of PLII and PLIII, attributable to their mercury-induced conformational changes. This modified binding to DNA changed the canonical protective role of PL proteins for DNA, as demonstrated in our previous work [28]. In conclusion, the alterations in PL proteins induced by mercury, shown in the present work, add information about the effects of heavy metals on this type of protein. Some of these effects, we had already demonstrated with other metals, such as cadmium and copper [3,4,27], but the results of the present work show that mercury is capable of inducing these effects on PL proteins, even with much lower exposure doses (pM). In addition, the results of this work provide further insights into the mechanisms of mercury toxicity on the reproductive system of *M. galloprovincialis*, even at mercury concentrations similar to those found in the Mediterranean Sea and in the oceans. [47,48]. As reported in this work, alterations in PL proteins after exposure of *M. galloprovincialis* to these HgCl_2_ doses alter their binding to DNA. This could adversely affect the structure of sperm chromatin, which is crucial for the swimming ability of *M. galloprovincialis* spermatozoa and for their ability to fertilize. After all, mercury is recognized as a male reproductive toxicant. In fact, in vitro studies have shown that this metal induces DNA breakage in spermatozoa and causes a decrease in sperm motility. Generally, Hg levels in semen are linked to abnormal sperm morphology, especially in head and middle defects, and low sperm viability [49]; indeed, the Hg levels in sub-fertile and infertile males are higher than in fertile males [50]. Moreover, in our previous paper, we reported the accumulation of mercury in exposed mussels after exposure to these HgCl2 doses [28]. The effects of mercury at low doses have also been found in other organisms. For example, exposure to low doses of Hg adversely affects sperm functions in rats through oxidative stress mechanisms [51], while it induces the inhibition of tyrosine phosphorylation of sperm proteins and alterations of the functional dynamics of buck spermatozoa [52]. In order to reveal possible alterations in the chromatin structure of spermatozoa from mussels exposed to these HgCl_2_ doses and to assess their fertilizing potential, our next objective will be to perform micrococcal nuclease experiments and in vitro fertilization tests. In addition, it will be interesting to assess the possible involvement of some particular amino acid able to bind Hg and to be responsible for the formation of PL protein aggregates and/or their conformational changes. Some studies have indicated that Hg binds histidines. For example, Stratton et al. 2016 [36] found that Hg binds two histidines of chymotrypsin, inducing aggregation of this protein. Secondary Hg^2+^-binding sites have been suggested to also contain histidines in alpha-lactalbumin [53]. Obviously, it cannot be excluded that, in interactions with mercury, there may be the involvement of other amino acids, such as arginines, in which PL proteins are particularly rich, as already reported in Tn10-en-coded metal-tetracycline/H1antiporter (TetA(B) [54], and this will be another future focus of our research. Finally, studies of PL proteins could be of interest in developing fast and reliable chromatin-based genotoxicity assays for biomonitoring programs for the assessment of heavy metal impacts and species management. 

## 4. Materials and Methods

### 4.1. Ethics Statement

This research was conducted on the marine invertebrate *M. galloprovincialis* (Lamarck 1819), which is not protected by any environmental agency in Italy. This study was performed in strict accordance with European (Directive 2010/63) and Italian (Legislative Decree n. 116/1992) legislation on the care and use of animals for scientific purposes.

### 4.2. Mussels Sampling and Exposure to HgCl_2_

In order to analyze the effects of HgCl_2_, adult mussels of *M. galloprovincialis* L (mixed sex) of average size shell lengths 4.93 ± 0.17 cm were kindly provided by Eurofish Napoli S.r.l. Bacoli, Italy. Mussels were exposed to 1, 10 and 100-pM HgCl_2_, as previously described when we exposed these organisms to other heavy metals [17], in laboratory plastic tanks (Ottavi, Cittadella, Italy) (36 × 22 × 22 cm). Each tank contained 6 L of 33‰ artificial sea water (ASW) with the following composition for 1 L: NaCl 29.2 g, KCl 0.60 g, MgCl_2_ 1.2 g, NaHCO_3_ 0.20 g and CaCl_2_ 1.08 g. In any tank were placed 13 mussels that were exposed to a single dose of HgCl_2_ for 24 h at 18 ± 1°C. Every 12 h, during exposure, the water and metal salts were changed, and dissolved oxygen and temperature were controlled at predetermined time intervals. The experiments were conducted in the winter period, January to February 2021. Tanks containing only ASW were used as a control for nonexposed mussels. For each condition, two tanks were used, for a total of eight tanks, as reported in Lettieri et al. 2019 [55].

### 4.3. Spermatozoa Sampling and Processing

Spermatozoa from male mussels were collected after 24-h exposure of the mussels to 1, 10 and 100-pM HgCl_2_. To obtain spermatozoa, the mussels were opened using a knife, being careful not to cut soft tissue. Then, after stimulating male gonads using a glass Pasteur pipette and seawater, gametes were obtained and examined by microscopic analysis to check that they were spermatozoa and assess sexual maturity based on a morphological and seminal analysis, as reported in Piscopo et al. 2018 [4]. Spermatozoa were collected as reported in Vassalli et al. 2015 [47]. In short, semen pooled from all male mussels contained in tanks corresponding to a given pM HgCl_2_ condition were centrifuged at 1000× *g* for 2 min at 4°C to discard debris. Supernatant obtained was centrifuged at 9000× *g* for 10 min at 4°C to collect spermatozoa in pellets of about 200 mg, which were recovered and stored at −80°C for further investigations.

### 4.4. PL Proteins from M. galloprovincialis Spermatozoa Extraction and Analyses

PL proteins were extracted from 10 sperm pellets corresponding to the exposure of mussels to a specific concentration of pM HgCl_2_, using 5% perchloric acid (PCA) as previously described [56]. In brief, spermatozoa pellets were homogenized in a potter with 15 mL of distilled water, and then, acid extraction with PCA was performed as reported by Vassalli et al. 2015 [47]. The sample containing PCA-soluble PL proteins was then extensively dialyzed against distilled water in order to remove all PCA and then lyophilized and stored at −80°C.

Two types of electrophoretic analyses were conducted for PL proteins: AU-PAGE, as previously described by Piscopo et al. 2018 [57], and SDS-PAGE, as previously described by Piscopo et al. 2020 [58], with a few modifications. In particular, the stacking gel was constituted by 5% (*w/v*) acrylamide (acrylamide/bis-acrylamide 29:1), and the separating gel was 18% (*w/v*) acrylamide (acrylamide/bis-acrylamide 29:1). At the end of the run both by AU-PAGE and SDS-PAGE, the gels were stained with Coomassie Brilliant Blue and acquired using a GelDoc system via Quantity One v.4.4.0 software (Bio-Rad, Hercules, CA, USA). The densitometric analysis of the gel bands was carried out using the software ImageJ ver. 1.50d (https://imagej.nih.gov/ij/ accessed on 15 April 2021), supported by the National Institute of Health (Wayne Rasband, National Institute of Mental Health, Bethesda, MD, USA). 

### 4.5. Plasmid DNA Preparation and Analysis

For the analyses of the DNA–PL interaction, pGEM3 plasmid DNA was used. This plasmid was prepared following the protocol described by Carbone et al. 2012 [59]. The quantification and quality of the plasmid DNA was evaluated with a UV-Vis spectrophotometer (NanoDropH ND-1000, Waltham, MA, USA), and the integrity of the DNA was analyzed on 1% agarose gels in 89-mM Tris-HCl, pH 8.0, 2-mM EDTA and 89-mM boric acid (TBE).

### 4.6. Turbidity Analysis of M. galloprovincialis PL Proteins

*M. galloprovincialis* PL proteins from nonexposed and HgCl_2_-exposed and bovine serum albumin (BSA) were analyzed at 0.85 mg/mL in Tris HCl 10 mM, pH 6.8, and the optical densities were evaluated at 420 nm (OD420). To the protein solution, increasing concentrations of HgCl_2_ (in the range from 0 to 400 µM) were added, and after each increase of HgCl_2_ concentration, absorbance at 420 nm was measured. The increase in absorbance (at 420 nm) was indicative of protein aggregate formation. In fact, we refer to this optical density as turbidity.

### 4.7. Fluorescence Spectroscopy Analyses

Fluorescence measurements of PL from nonexposed (control) and exposed mussels to HgCl_2_ were performed using a PerkinElmer (Waltham, MA, USA) luminescence spectrometer LS-55 by using 1 mL of PL solution 1 mg/mL in water in the presence of 5-μM ANS in a 1-cm optical path cuvette. After excitation at 350 nm, fluorescence spectra were obtained in the emission range of the wavelengths from 410 to 600 nm. Three measurements were performed for each sample: PL from nonexposed (control) and exposed mussels to 1 pM, 10 pM and 100-pM HgCl_2_. Data were analyzed with the software QtiPlot 1.0.0 rc13 (ver. 5.12.8). 

### 4.8. Effect of PL Addiction from HgCl_2_-Exposed Mussels on the DNA Absorption Spectrum

To evaluate the DNA–PL protein interaction, absorption spectra of DNA following the adding of PL from 1 pM, 10 pM, and 100-pM HgCl_2_-exposed mussels were conducted. For DNA spectra, 1-µg plasmid DNA in 400 µL of 1X TEB was used. Spectra were recorded in the range 200–300 nm. The absorption spectrum of plasmid DNA alone and those relative after the addition of increasing amounts of PL in the 0.5–1–1.5–2–3 protein/DNA ratios (*w/w*) were determined.

### 4.9. Preparation of M. galloprovincialis Sperm Nuclei and Salt-Induced Release of Nuclear Proteins

For the preparation of sperm nuclei, the procedure reported in Olivares and Ruiz 1991 [60] was followed. The release of sperm nuclear basic proteins was done following the protocol described by De Guglielmo et al. 2018 [43] using increasing NaCl concentrations: 0.2 M, 0.4 M, 0.5 M, 0.65 M, 0.8 M, 1 M, 2 M, 3 M and 4 M. For each suspension, incubation at 4°C for 30 min and then centrifugation at 13,000× *g* for 30 min was performed. Sperm nuclear basic proteins were extracted from supernatants with 0.2-N HCl (final concentration). The samples were incubated for 16 h at 4 °C and then centrifuged for 30 min at 13,000× *g*. The supernatants obtained were widely dialyzed with distilled water. Four micrograms of proteins in each sample were examined by AU-PAGE (in accordance with Fioretti et al. 2012 [61]) to assess the stepwise release of these proteins from sperm DNA as a function of the NaCl concentration. A sample containing a PL protein and core histones was obtained by extraction with 0.2-N HCl from *M. galloprovincialis* spermatozoa using the same method described for PCA extraction. This sample served as a reference in the electrophoretic analysis for protein band quantification.

### 4.10. Statistics Analysis

The data were analyzed using one-way ANOVA. Tukey’s test and Student’s *t*-test were used to compare the means between the groups. Values were considered significant when *p* < 0.05. Statistically significant differences were defined at the 95% confidence interval. Data were shown as the mean ± SD.

## Abbreviations

ASW	Artificial sea water
AU-PAGE	Acetic acid-urea polyacrylamide gel electrophoresis
SDS-PAGE	Sodium dodecyl sulphate–polyacrylamide gel electrophoresis
EDTA	Ethylenediaminetetraacetic acid
EMSA	Electrophoretic mobility shift assay
PCA	Perchloric acid
PL	Protamine-like
SNBP	Sperm nuclear basic protein
TBE	Tris/Borate/EDTA
OD	Optical density
